# Harassment as a consequence and cause of inequality in academia: A narrative review

**DOI:** 10.1016/j.eclinm.2022.101486

**Published:** 2022-06-03

**Authors:** Susanne Täuber, Kim Loyens, Sabine Oertelt-Prigione, Ina Kubbe

**Affiliations:** a University of Groningen, Groningen, The Netherlands; b Utrecht University, Utrecht, The Netherlands; c Radboud University Medical Center, Nijmegen, The Netherlands; d Bielefeld University, Bielefeld, Germany; e Tel Aviv University, Tel Aviv, Israel

**Keywords:** Higher education, Sexual harassment, Inequality, Intersectionality, Integrity, Corruption

## Abstract

A growing body of literature suggests that over the past 30 years, policies aimed at tackling harassment in academia have had little discernable effect. How can this impasse be overcome to make the higher education sector a safe space for everyone? We combine the areas of harassment and inequality, intersectionality, policy-practice gaps, gender sensitive medicine, as well as corruption and whistleblower processes to identify lacunae and offer recommendations for how to apply our recommendations in practice. We have been searching the most influential, relevant, and recent literature on harassment and inequality in our respective fields of expertise. By studying conceptual overlaps between the different fields, we were able to create insights that go beyond the insights of the most recent reviews. Our synthesis results in three concrete recommendations. First, harassment and inequality are mutually reinforcing. Failure to adequately tackle harassment contributes to perpetuating and reproducing inequality. Further, the intersectional nature of inequality has to be acknowledged and acted upon. Second, enforcing anti-harassment policies should be a top priority for universities, funders, and policymakers. Third, sexual harassment should be treated as institutional-level integrity failure. The higher education sector should now focus on enforcing existing anti-harassment policies by holding universities accountable for their effective implementation - or risk being complicit in maintaining and reproducing inequality.

**Funding:**

We have received no funding for this research.

## Introduction

Over the past years, a number of highly influential reviews about sexual harassment have demonstrated its pervasiveness in academia at all levels of the hierarchy.^1^, ^2^, ^3^ Harassment, defined as “a range of unacceptable behaviours and practices, or threats thereof, whether a single occurrence or repeated, that aim at, result in, or are likely to result in physical, psychological, sexual or economic harm”,^4^ represents a widespread occupational hazard.^5^^,^^6^ A more recently used term is gender-based violence (GBV), which refers to “violence directed against a person because of that person's gender or violence that affects persons of a particular gender disproportionately”.^7^ It includes forms of violence, violations, and abuse that can be physical, sexual, psychological, symbolic, economic, and generally cause suffering to those targeted.^7^ We use these terms interchangeably throughout the paper. Sexual harassment has enormous detrimental effects on targets’ mental and physical health, their careers, and their livelihoods.^8^ Despite the well-established insights about the widespread experience and debilitating consequences of sexual harassment, anti-harassment and non-discrimination policies are oftentimes ineffective.^1^ Experts in the field of higher education observe that over a time span of 30 years, no discernable progress has been made, with scholars still pointing to the same lacunae and recommending the same measures.^2^ In fact, scholars point to the situation getting worse, partly because the small steps towards more equality prompt disproportional backlash.^9^, ^10^, ^11^ This is illustrated by the following quote by a researcher interviewed for a recent Nature survey on discrimination, who says “Everybody talks about equality in science, but it doesn't actually happen,” ... “There are so many articles, so much discussion, but over my 30 years it's gotten worse”.^12^

With this review, we aim to provide novel insights for how to overcome the observed impasse and move towards effective anti-harassment policies and a more equal, diverse and inclusive higher education sector. We here combine our respective areas of expertise - harassment and inequality, intersectionality, policy-practice gaps, gender sensitive medicine, as well as corruption and whistleblower processes - to provide a fresh point of view, identify lacunae and promising ways forward. We point to three important yet currently underacknowledged issues that we deem essential for improving policies and advancing science. These concern the fact that inequality and harassment are mutually reinforcing powers, with inequality enabling harassment, and harassment perpetuating and reproducing inequality. A second issue concerns the question of who the harassers are and why they harass, a question that is hardly ever asked due to the strong overall focus on the targets of harassment and inequality. Finally, we contend that inequality and harassment amount to integrity failures beyond the individual perpetrator. Rather, they point to institutional level integrity failures akin to corruption, because they enable phenomena such as nepotism, malpractice, breach of contract, personal advantages and unfair competition. Accordingly, we propose that strong sanctioning is warranted where universities fail to effectively fight harassment and discrimination. We derive concrete recommendations for university leadership, funders, and policymakers based on our review. We further hope to stimulate new research employing process-oriented, longitudinal, and multidisciplinary approaches to study the relationship between harassment, inequality, and integrity failures in higher education.

### Search strategy and selection criteria

References for this review were identified by a general search for articles related to harassment, inequality, and corruption in higher education and public governance. We prioritized literature that could span the boundaries between our adjacent fields of expertise, in particular concerning harassment and inequality, intersectionality, policy-practice gaps, gender sensitive medicine, as well as corruption and whistleblower processes. We further prioritized literature from the past five years starting 2018, as this signifies the time when the first relevant review was published.^3^ Different from traditional systematic reviews, this method mainly relies on our insights as experts in the respective field. We have chosen this approach for two reasons: First, within all the fields in focus, research has been accumulated on the question of causes and consequences of harassment and inequality. Reviewing all the available literature of the fields separately is not feasible. However, recent systematic reviews^1^, ^2^, ^3^ partly touched on several of the disciplines we were aiming to integrate, and therefore offered a feasible starting point particularly regarding the higher education sector. Second, an attempt to integrate and synthesize the insights of our respective fields of expertise to explain why anti-harassment policies are ineffective and inequality prevails in academia, has not been done before – hence, we have no prior studies to review that would tap into this specific challenge. In fact, we hope for our narrative review to stimulate such studies in the future.

Taken together, we built a narrative review based on a synthesis of our respective fields of expertise to confront the unresolved problem of harassment in higher education. Focusing on the most relevant and recent scholarship within these fields of expertise, we organize the review around the following topics: the relationship between harassment and inequality, characteristics and motives of harassers, and the role that universities play in enabling harassment and reproducing inequality. We derive recommendations for three concrete areas for action based on the review.

Inequality enables harassment. Acker's^13^ seminal concept of inequality regimes is instrumental for understanding how harassment is enabled and perpetuated by existing inequality in academia. Inequality regimes refer to the “interlocked practices and processes that result in continuing inequalities in all work organizations” (p. 441). These practices and processes are often invisible and informal, and form one key reason for the ineffectiveness of anti-harassment policies. Specifically, the formal rules and regulations associated with equality, diversity and inclusion in academia clash with informal practices and processes that keep benefitting certain groups of people and disadvantage others. Organizational cultures that are conducive of harassment thus play a key role in reproducing inequality. These are cultures characterized by masculinity, competitiveness, and individualism, such as the police force^14^ and academia.^15^ For example, of the more than 4000 academics responding to a Wellcome Trust survey, 61% reported witnessing bullying or harassment, and 43% said they had experienced it.^16^ The perpetrators are overwhelmingly white male supervisors abusing their position of power.^17^ Members of underrepresented groups who face intersectional disadvantages, such as women in precarious employment conditions, or foreign female scholars, are targeted by GBV more.^8^^,^^18^, ^19^, ^20^

Attesting to the undoing of anti-harassment policies by informal and covert inequality regimes, Healy et al. (21, p. 1752) suggest that “informal interactions may thus be of greater significance in the daily experience of work than formal policy statements”. To overcome the resulting policy-practice gap, universities would have to rigorously enforce their zero tolerance policies, but the contrary is the case: Reporters of misconduct are blamed, silenced, gaslighted, and face fierce retaliation,^2^^,^^21^ while the harassers are typically protected by HR officers, higher management and university leadership.^22^ Harassment consequently often goes on for decades with the knowledge of higher management; in a recent case of Harvard University, management only acted 20 years after the first allegations against a chair holder surfaced.^23^ In addition, harassment is often not prosecuted due to corrupt activities including favoritism, conflict of interest or bribes as a form of hush money. Illustrating this, a BBC investigation^24^ found that, since 2016, U.K. universities had spent an aggregate sum of £1.3m to silence student grievances, including sexual assault and bullying, with non-disclosure agreements (NDA's). A hearing in the Irish parliament revealed that higher education institutions used public money in NDAs to silence victims of discrimination and sexual harassment.^25^ Not surprisingly, a cross-sectional global survey among more than 2000 respondents working at academic science institutions^26^ revealed that only 8% of those who reported bullying and abuse thought the process was unbiased and fair. Together, the individual costs of reporting and the institutional protection of the harassers contribute to massive underreporting of harassment in academia.^8^ In turn, underreporting contributes to the preservation of organizational cultures that enable harassment in the first place.^1^

But not only open discrimination and harassment contribute to ongoing inequality in academia. Research offers accumulating evidence for processes of discounting and devaluing of performance, achievements, and contributions of underrepresented groups in academia. Ma and colleagues^27^ show, for instance, that prize-winning female scientists receive less money, less public attention, and less career advancement compared to their male counterparts. Similarly, when examining the near-complete population of approximately 1.2 million U.S. doctoral recipients from 1977 to 2015, Hofstra and colleagues^28^ show that underrepresented groups produce higher rates of scientific novelty. However, their novel contributions are devalued and discounted, consequently not translating into successful academic careers – a phenomenon coined the diversity-innovation paradox.^28^

In sum, the enabling of harassment in academic organizational culture systematically puts down and pushes out members of underrepresented groups, in particular those facing intersectional disadvantages. In contrast to this, intersectional inequality and the associated disproportional exposure to harassment is still neglected by most policy measures, which tend to focus on the singular aspect of gender, thereby benefitting a narrow subpopulation of white, middle-class, cis-gender women.^29^ The perpetuation and reproduction of (intersectional) inequality in academia also contribute to the making of new harassers through dynamics that are not yet well-understood, but require more attention by scholars and policy makers. We review recent insights into these dynamics below.

### Harassers perpetuate and reproduce inequality

So far, the bulk of research and policy making focuses on the targets of harassment. We propose that the perpetrators and their institutional enablers require more attention. Harassment, as is increasingly well-understood, helps those perpetrating it to advance their careers at the cost of (multiply) underrepresented groups. In occupational contexts, sexual harassment is often used to control women, ultimately aiming to maintain the gender hierarchy.^30^ This aspect of harassment – how it can be instrumental to further the careers of those engaging in it – is not yet central to research and policy, but it should be. This requires attention to specific forms of harassment in universities, such as the sabotaging of academic careers.^31^ These include the denial of promotion, exclusion from informal knowledge and networks, lack of opportunities for professional development, greater teaching load and less research time, and so on.^15^^,^^21^ By sabotaging the careers of members of (multiply) minority groups, harassers further their own careers, rise into powerful positions, and have ample opportunity to perpetuate and reproduce the inequality that enables harassment. Harassers also often make new harassers and feed them into the system as their “crown princes”.^17^^,^^31^ Furthermore, empirical research indicates a strong link between corruption and inequality, in particular gender equality, based on the fact that corrupt practices affect women more often.^32^^,^^33^ Often harassers use a corrupt network that mainly consists of men and protects and hides their illegal actions.^34^ Thus, harassment can go hand in hand with corrupt forms such as favoritism and sextortion that women suffer from more often, in particular in the educational sector.^35^^,^^36^ Yet, these specific links are still not well researched.

One important route forward is therefore to stop depicting harassment as a minor personality flaw, as is often the case when “star academics” are involved. Note that we do not suggest that the majority of ‘star academics’ or university leadership are harassers. However, research and reports increasingly suggest that harassers are more likely to occupy leadership positions in academia, because of certain personality traits such as narcissism, psychopathy and Machiavellianism.^37^ These personality traits make academics more prone to engage in research misconduct such as fabrication and falsification of data and plagiarism (FFP; 39), which seems to amount to a “survival benefit” in academia.^37^ Forster and Lund^38^ propose that psychopathic traits may be an advantage for people aspiring to become a leader in higher education, as the high competitiveness characterizing the sector matches personality traits such as boldness and dominance, meanness, and disinhibition. It is not surprising, then, that such personality traits play a role not only in research misconduct, but in harassment, too. For instance, clear theoretical links are reported between psychopathy and bullying.^39^ In their description of what typical psychopathic traits in higher education look like, Forster and Lund^38^ refer to leaders who routinely overstate their own achievements while belittling those of junior faculty, or the planting of false stories to harm the reputation of colleagues, or faculty members who publicly ridicule, slander, or besmirch the achievements of colleagues, and so on.

Indeed, the majority of the scientific sabotaging accounts shared in recent reports from Dutch academia,^15^^,^^21^ match these descriptions. The sabotaging of scientific careers of female scholars entailed using vague and ever-changing performance criteria to deny promotion, exclusion from professionalization opportunities to justify denying tenure and professorships, and quite obvious discrimination based on sex, age, culture and religious beliefs. Interestingly, interviewees – in particular foreign female scholars – converge in their reports of such harassment starting or worsening after they began to establish excellent track records of publications and/or funding. Interviewees pointed to the possibility that their excellent performance triggered backlash and harassment because it frustrated their superiors’ intention to promote their “crown princes” in a seemingly legitimate manner. Other colleagues would question the promotion of visibly lower-performing proteges of chair holders when female colleagues secured substantial funding, for instance.^21^ Such a pattern, if validated by other studies, would align well with the notion that harassment benefits the careers of the harassers and hence serves as a competitive advantage, albeit in an unfair competition. A number of recent scholarly works support this proposition.^17^^,^^40^, ^41^, ^42^, ^43^

Recent research employing an evolutionary lens on harassment^44^ demonstrated that when females enter male-dominated hierarchies, which are usually based on clientelistic networks,^33^^,^^45^ it is the poor performing males who engage in hostile behavior. The authors suggest that this is because these males face the highest risk of losing their status when highly qualified and skilled females enter the working environment. Research on the effects of zipper quota in a political party supports this proposition. Where the zipper quota increased female representation the most, the competence of male politicians increased the most – because less competent male politicians resigned. The authors accordingly refer to quota as causing a crisis of mediocre men.^40^ In sum, the evidence suggests that, on the one hand, harassment is incentivized in academia because it benefits the harassers’ career advancement,^17^^,^^41^ and that, on the other hand, harassment might be a response to changing power configurations particularly among the less qualified, who would not easily achieve and hold on to a high-status position in academia if career advancement was indeed linked to meritocratic principles.

### Universities enable harassment and inequality reproduction

**Figure 1:**
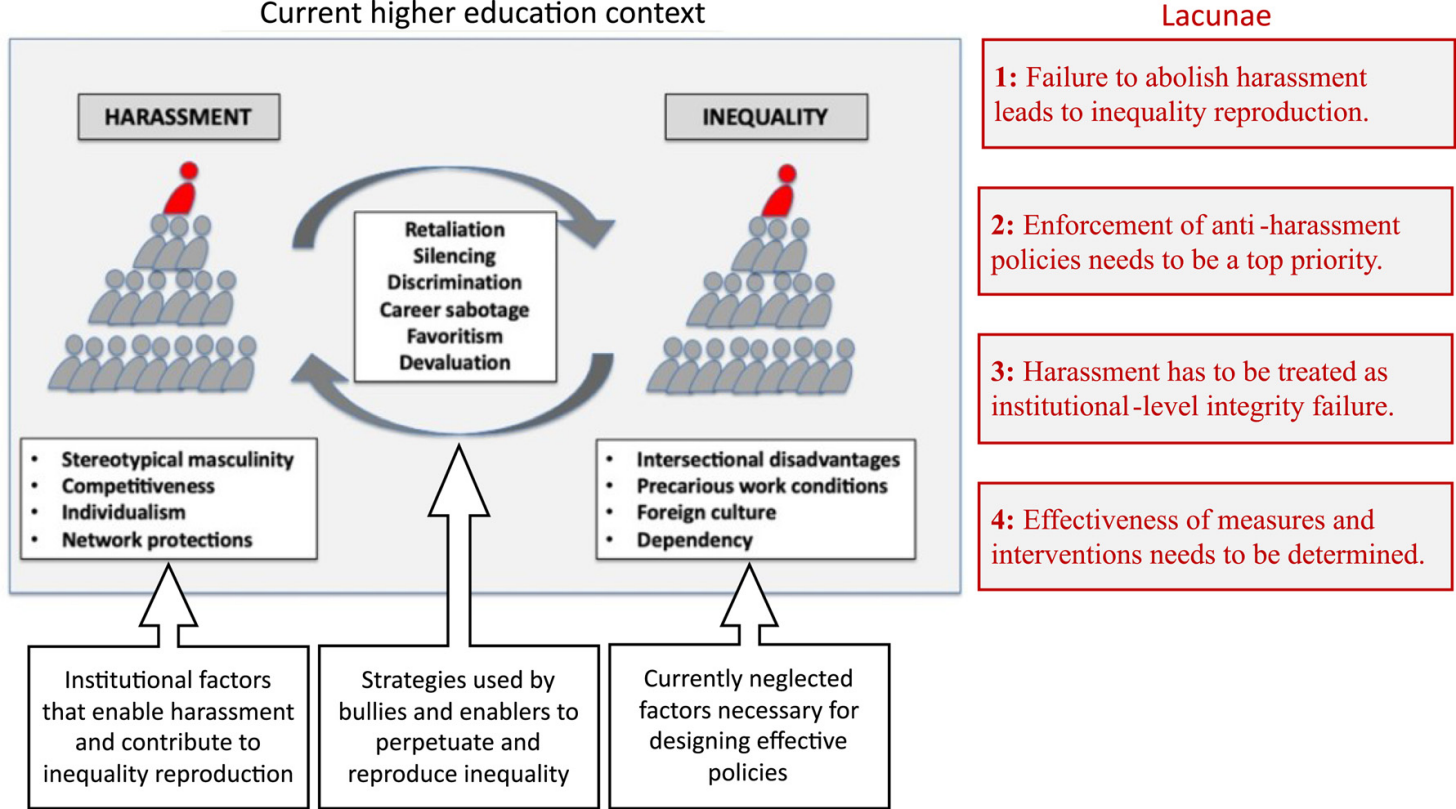
An illustration of the different actors and forces affecting harassment and inequality in academia. Institutional factors, strategies used by bullies and enablers, as well as currently neglected factors provide insight into the most pressing current lacunae. If unaddressed, these lacunae contribute to the mutual perpetuation and reproduction of inequality and harassment in academia.

It is important to keep in mind the interaction of personality traits of harassers and the organizational environment that enables them. Forster and Lund^38^ point to the relative lack of institutional oversight and safeguards in higher education organizations, which facilitate “academics with psychopathic characteristics who have risen to senior management and administrative roles in which they can act out their toxic tendencies under the guise of the legitimate authority of the organizations that employ them” (p. 24). Indeed, harassers typically do not operate alone. Accumulating research demonstrates how harassers are facilitated and protected by a large network of HR-lawyers, HR-officers, and higher management.^46^ Several authors have demonstrated that colleagues of the perpetrators and HR advisors enable sexual harassment to go unsanctioned by forcing targets into “reluctant acquiescence”.^21^^,^^22^^,^^47^ Thus, employees cannot count on the enforcement of anti-harassment policies, especially not in sectors that are notorious for power imbalances such as the police force^48^^,^^49^ or academia.^15^^,^^21^ Harassers and their enablers often employ a strategy whereby the harassment is denied, the victim attacked, and victim and offender roles reversed (Deny-Attack-Reverse Victim and Offender, DARVO; 51). The social dynamics surrounding sexual harassment result in fear cultures in which silence is safer than speaking up,^46^ because “powerful norms of censorship” (52, p. 1043) are implicitly reproduced. While research demonstrates that institutions might retaliate against whistleblowers because they feel betrayed by their speaking up,^50^ reporters of harassment can themselves also feel betrayed by the institution, for instance when a victim is being silenced to protect a harasser. Such perceptions of institutional betrayal often exacerbate targets’ traumatic experiences.^51^ Against this background, it is not surprising that legal scholars contend that, when the Universal Declaration of Human Rights is considered, academic bullying is a human rights violation because it denies dignity to those targeted.^52^ This aligns with the definitions used by influential organizations such as the United Nations and the European Commission, which conceive of GBV as human rights violations. Given the havoc that harassment spreads, universities have to step up their game and rigorously enforce the anti-harassment and non-discrimination policies they have developed over the past 30 years. The review presented here suggests that higher education institutions, even if inadvertently, incentivize harassment with academic success, near-complete inviolability and protection. Figure 1 summarizes the findings. If higher education institutions fail to intervene effectively with these dynamics, then other stakeholders have to make sure that harassers and institutions are held to account. Below, we present three ways forward.

## Conclusions

Our review offers three essential insights for future research and policymaking to effectively tackle harassment and GBV in academia. First, harassment and inequality mutually reinforce one another. Where existing inequality regimes enable harassment, failure to abolish harassment contributes to perpetuating and reproducing this inequality. However, the intersectional nature of inequality is not sufficiently acknowledged and acted upon yet. Reflecting on what intersectionality means today, Kimberlé Crenshaw, who coined the term thirty years ago, says “It's basically a lens, a prism, for seeing the way in which various forms of inequality often operate together and exacerbate each other. We tend to talk about race inequality as separate from inequality based on gender, class, sexuality or immigrant status. What's often missing is how some people are subject to all of these, and the experience is not just the sum of its parts.”^53^ University leadership and management need to educate themselves about intersectional inequality and how it might undermine the effectiveness of anti-harassment and non-discrimination policies. Acknowledging that employees with intersecting forms of inequality are more vulnerable to becoming targets of harassment is important. Interventions should be designed with intersectionality in mind and evaluated regarding their effectiveness for multiply underrepresented groups, instead of centering around white, middle-class, cis-gender women.^29^ Neglecting intersectional inequality undermines anti-harassment policies because it leads to unintended side effects of such policies^54^^,^^55^ and can even harm those they intended to serve through backlash.^9^, ^10^, ^11^^,^^56^^,^^57^ In addition, intersectional inequality harms science itself because some topics that are traditionally studied more by scholars from marginalized groups receive lower citation rates and become systematically less studied.^58^ These authors showed that if “the author distribution over the last 40 y would have matched the 2010 US Census, there would have been 29% more articles in public health, 26% more on gender-based violence, 25% more in gynecology and in gerontology, 20% more on immigrants and minorities, and 18% more on mental health” (60, p. 6).

Second, enforcing anti-harassment policies needs to be a top priority for universities, funders, and policymakers. Other scholars have recently advocated for such a multi-stakeholder approach, most notably by introducing the Framework for Coordinated Global Actions To Diminish Academic Bullying.^59^ Hollis^52^ argues that workplace bullying should constitute a human rights violation, and that universities are particularly important in fighting harassment, because of the high prevalence of workplace bullying in higher education. Scholars recommend that university policy makers proactively confront resistance against the implementation and enforcement of anti-harassment measures among those with privilege and power.^60^ Universities need to establish processes that bypass individual biases and personality traits associated with harassment, given that particularly the “mean and mediocre” appear to abuse the lack of accountability and oversight in the higher education system.^17^^,^^39^^,^^41^ That means that universities have to be held to account if they fail to effectively address harassment at their institution. Probably in response to universities’ persistent struggle to enforce anti-harassment policies, funders, legislators and politicians in a number of countries are currently taking measures themselves. For instance, the NIH, the largest funder in the U.S., removed more than 70 lab heads from grants after probing more than 300 complaints of harassment, including sexual harassment and racial discrimination.^61^ The Wellcome Trust, a major UK research funder, has vowed to pull grants from universities that fail to comply with their misconduct policy, including failure to report harassment.^62^ Similarly, the Irish minister for Further and Higher Education issued his opposition to universities’ use of public money for NDAs that silence victims of sexual harassment or bullying in Irish higher education institutions, stressing that “the use of non-disclosure agreements runs contrary to the values of transparency, consistency and integrity”.^25^ In addition to enforcing, inclusivity can be incentivized, which is a strategy followed by the European Commission. The commission has made it mandatory for public bodies, research organizations and higher education establishments to have Gender Equality Plans in order to get access to Horizon Europe funding, starting in 2022.^63^

This leads to the third point, namely that harassment and GBV should be treated as institutional-level integrity failures. Harassment is almost always enabled and facilitated by underlying structures of inequality, such as large power imbalances. Higher education institutions are thus complicit in harassment and inequality reproduction.^38^^,^^64^, ^65^, ^66^ The blaming and silencing of victims, gaslighting and retaliating against reporters of harassment amplify this integrity failure and its consequences. As of yet, bullying complaint procedures are often distinct from whistleblowing arrangements, based on the argument that the former does not involve the public interest.^67^ Yet, this avenue might be beneficial for the complainant, because the allegation is then “treated as a protected disclosure” resulting in “a degree of protection” against unfair dismissal and compensation (70, p. 121). Moreover, workplace bullying complaints as whistleblowing allow for a more systemic approach by placing responsibility at the organizational level rather than only focusing on the interaction between harasser and harassed person, which could unveil institutional root causes of the harassment.^68^^,^^69^ Combined with confidentiality in the reporting procedure, an external whistleblowing trajectory could be particularly fruitful, because it would help to avoid retaliation by those involved. Implementing whistleblower arrangements might thereby prevent additional trauma that targets of harassment often experience because of their institutions’ failure to adequately respond to their complaints.^51^ Highlighting the associated integrity failure, the British Minister of State for universities informed all higher education providers in the U.K. that “hiding workplace harassment or withholding details of complaints is unacceptable”.^70^ Indeed, universities’ failure to effectively implement and enforce anti-harassment policies enables nepotism, malpractice, breach of contract, clientelism and unfair competition – all with public money. Favoritism, for instance, is an established form of corruption that does manifest even without monetary rewards.^71^ Pointing to the relevance of gender equality as an important tool to disrupt and prevent corruption, the United Nations Office on Drugs and Crime^36^ notes that existing patronage networks are predominantly male. Considering universities’ failure to enforce anti-harassment policies through the lens of corruption might offer new tools for enforcement and sanctions.

Table 1 provides an overview of these recommendations. A recent case^23^ illustrates that the higher education sector is starting to face fall-outs from neglecting the associations between harassment, power abuse and corruption. The federal lawsuit against Harvard professor John Comaroff shows universities’ complicity and that targets are willing to sue. The first sentence of the federal lawsuit against Harvard is “This is a case about Harvard's decade-long failure to protect students from sexual abuse and career-ending retaliation.”^72^ Indeed, commentators call for holding the internal committees accountable for failing to protect targets of harassment, both for their failure to stop the harasser and for “ganging up against the people filing the complaints or helping cover up these atrocious behaviors”.^72^

**Table 1:** Recommendations to effectively tackle harassment and gender-based violence (GBV) in academia.

Current lacunae	Recommendations for specific actors
*1. Failure to abolish harassment contributes to perpetuating and reproducing inequality, especially when the intersectional nature of inequality is not sufficiently acknowledged or acted upon yet.*	University management • Acknowledge that employees with intersecting forms of inequality are more vulnerable to becoming targets of harassment. • Design and evaluate interventions with intersectionality and underrepresented groups in mind to avoid unintended side effects of anti-harassment policies Researchers • Cite scholars from marginalized groups who study intersectional inequality more frequently. • More scholars from non-marginalized groups should include an intersectional perspective in their work.
*2. Enforcing anti-harassment policies is not yet a top priority for universities funders, and policymakers.*	University management • Proactively confront resistance against the implementation and enforcement of anti-harassment measures among those with privilege and power. • Establish processes that bypass individual biases and personality traits associated with harassment, given that particularly the “mean and mediocre” appear to abuse the lack of accountability and oversight in the higher education system. Funders and policymakers • Hold universities accountable if they fail to effectively address harassment at their institution. • Take decisive measures in response to harassment in academia, such as pulling grants from universities that fail to comply with their misconduct policy. • Design incentives for inclusivity, for example by making it mandatory for public bodies, research organizations and higher education establishments to have Gender Equality Plans.
*3. Harassment and GBV are not yet treated as institutional-level integrity failures.*	Policymakers and university management • Install external whistleblowing trajectories to minimize retaliation and guarantee confidentiality. • Guarantee protection and confidentiality for those who report harassment and bullying as victims, for example by making it part of whistleblowing arrangements. • Treat workplace bullying complaints more systemically and make serious attempts to unveil institutional root causes of the harassment.
*4. Effectiveness of the suggested measures cannot be determined yet.*	University management and researchers • Evaluate whether the measures suggested in the literature effectively reduce harassment and increase equality in academia. • Exchange experiences and expertise at the level of departments, institutes, and universities to country- and discipline-levels. Policymakers • Keep universities engaged in their efforts, for instance by monitoring the progress and offering support with concrete policies and interventions.

It is yet too early to determine whether the reviewed measures to increase awareness and accountability are effective in reducing harassment and increasing equality in academia. Evaluations of the effectiveness of such measure are urgently warranted and might profit from pooling experiences at the level of departments, institutes, and universities to country- and discipline-levels. In addition, and tying in with a multi-stakeholder approach,^60^ the higher education sector must keep universities engaged in their efforts, for instance by monitoring the progress and offering support with concrete policies and interventions.

## Conclusion

Our review stresses the need for enforcement and sanctions to fight the harassment epidemic in academia. Most countries have sufficient legal provisions addressing workplace violence and harassment in national occupational safety and health and labour laws and regulations^73^^, Appendix II^. The problem is the enforcement of these legal provisions. For instance, reflecting on the potential impact of making coercive control a criminal offence in Scotland, Burman and Brooks-Hay^74^^, p. 78^ concluded that “Legislative change cannot on its own lead to improvements. Whatever laws we have will be only as effective as those who enforce, prosecute and apply them. Improving these practices – through education, training and embedding best practice and domestic abuse expertise – is likely to be more effective than the creation of new offences alone.” This sentiment is echoed in Bondestam and Lundqvist^8^’s systematic review of harassment in higher education. The authors conclude that “There is actually nothing to suggest that further efforts to strengthen the impact of policy on sexual harassment (information, communication, revising policies) will change underreporting, policy awareness, or reporting behaviour as such” (p. 406). Thus, rather than designing new policies, the higher education sector should focus its efforts now on enforcing existing anti-harassment policies by holding universities accountable for their effective implementation or risk being complicit in maintaining and reproducing inequality.

## Contributors

Susanne Täuber, Kim Loyens, Sabine Oertelt-Prigione, Ina Kubbe. [Conceptualization: ST, KL, SOP, IK; Identification of relevant literatura per field and synthesis: ST, KL, SOP, IK; Preparation of initial draft: ST; Review and editing: IK, SOP, KL, ST; Visualization: SOP; Table: KL]

## Declaration of interests

Susanne Täuber is a member of the Dutch Advisory Committee Diverse and Inclusive Higher Education and Research, which provides advice on promoting an inclusive, diverse and safe learning and working environment within the field of higher education and in scientific research and a member of the advisory board at the Academic Parity Movement, a nonprofit organization dedicated to addressing academic discrimination, violence and incivility.

Sabine Oertelt-Prigione was a member of the EU Expert Group Gendered Innovations 2 and is the chairwoman of the current EU Expert Group Gender and COVID-19. She received a grant from the German Ministry of Health, Stichting Achmea Slachtoffers en Samenleving, Dutch Heart Foundation.

Kim Loyens was part of the organizing committee of the International Whistleblowing and Research Network conference in Utrecht (2019).

Ina Kubbe has not conflicts to report.

## Funding

We have received no funding for this research.
